# Losing the identity of a hockey player: the long-term effects of concussions

**DOI:** 10.2217/cnc-2019-0014

**Published:** 2020-05-11

**Authors:** Åsa Engström, Eija Jumisko, Pashtun Shahim, Niklas Lehto, Kaj Blennow, Henrik Zetterberg, Yelverton Tegner

**Affiliations:** 1 Division of Nursing, Department of Health Science, Luleå University of Technology, Luleå, Sweden; 2 Development Manager, Lapland University of Applied Sciences, Rovaniemi, Finland; 3 Clinical Neurochemistry Laboratory, Institute of Neuroscience & Physiology, Sahlgrenska Academy at University of Gothenburg, Sahlgrenska University Hospital, Mölndal, Sweden; 4 Division of Medical Sciences, Department of Health Science, Luleå University of Technology, Luleå, Sweden; 5 Department of Psychiatry & Neurochemistry, Institute of Neuroscience & Physiology, the Sahlgrenska Academy at the University of Gothenburg & Clinical Neurochemistry Laboratory, Sahlgrenska University Hospital, Mölndal, Sweden; 6 Department of Neurodegenerative Disease, UCL Queen Square Institute of Neurology, Queen Square & UK Dementia Research Institute at UCL, London, UK; 7 Division of Medical Sciences, Department of Health Science, Luleå University of Technology, Luleå, Sweden

**Keywords:** concussion, hermeneutic phenomenology, hockey player, identity, injury, qualitative research

## Abstract

**Aim:**

To describe what suffering multiple concussions meant for former semi-professional or professional hockey players who were forced to end their career.

**Results:**

Nine former Swedish hockey players, who once played on national or professional teams were interviewed. The interviews were analyzed with reference to hermeneutic phenomenology to interpret and explain their experiences. The theme of losing one’s identity as a hockey player was constructed from five subthemes: being limited in everyday life, returning to the hockey stadium as soon as possible, forming a post career identity, lacking understanding and support, and preventing injuries by respecting other players.

**Conclusion:**

The former hockey players struggled with developing their off-the-ice identities and with finding other sources of meaning for their lives.

## Lay abstract

Despite considerable attention to improving the initial management of concussions suffered by hockey players, few studies have examined their long-term effects. In response, the study reported here aimed to describe what suffering multiple concussions meant for former semi-professional or professional hockey players who were forced to end their career. Nine former Swedish hockey players, who once played on national or professional teams were interviewed and the interviews were analyzed in order to interpret and explain their experiences. The overall theme was formulated as losing one’s identity as a hockey player. In conclusion, the former hockey players seem to struggle with developing their off-the-ice identities and with finding other sources of meaning for their lives.

Playing ice hockey poses a persistent risk of injury due to the sport’s forceful, body-to-body contact, especially when played on ice rinks encompassed by rigid walls. Despite using protective equipment, ice hockey players continue to experience a high injury incidence [1,2], including an increased number of concussions [3]. Following frequent news media reports [4] of the alarming, long-term and often debilitating effects of concussions and other head injuries suffered by ice hockey players [5–7], concussions have become a major source of concern for individuals involved in the sport [8,9].

As stated in the Consensus statement on concussion in sport, a sports-related concussion is a traumatic brain injury (TBI) induced by biomechanical forces resulting from a direct or indirect blow to the body – often to the head, neck or face – and subsequently transmitted to the brain [10]. Typically, a concussion initiates a rapid onset of signs, symptoms and forms of neurological impairment that often resolve spontaneously. However, it may result in neuropathological changes and the acute symptoms reflect a functional disturbance in the brain [10]. Consequently, diagnosing a concussion requires a multifaceted approach to evaluate possible forms of impairment in several domains of neurological function [11]. Typically, cognitive dysfunction and symptoms follow a similar course of recovery, although symptomatic recovery often precedes cognitive recovery [12,13]. Nevertheless, recovery after a concussion does not follow an established timeline [14]. A history of prior concussion may increase the risk of subsequent concussions [15,16], especially if the athlete returns to play prematurely [6]. Multiple concussions may lead to chronic neurobehavioral deficits and a progressive neurodegenerative disease termed chronic traumatic encephalopathy [17]. The latest guidelines by the Concussion in Sport Group [10] state that: “*an athlete suspected of suffering a concussion should be immediately removed from the field of play and assessed by a healthcare professional experienced in concussion management using the the Sport Concussion Assessment Tool 5 (SCAT5)* [18], *which is a side line assessment tool*’.”

The athlete should initially take cognitive as well as physical rest and not directly return to the sport until they have gradually been through rehabilitation [10]. These guidelines aim to improve the initial management of concussions and the risk for chronic sequelae. Giza *et al.* [19] suggested that prolonged total restriction of cognitive brain activity is not ideal, not even for an injured brain. Instead an initial brief rest, followed by controlled reintroduction of cognitive activities, no risky physical activities and, eventually, a return to normalcy is suggested.

Most athletes who sustain a concussion will be able to return to activity within 14 days, but approximately 20% develop post concussive symptoms [20]. Post concussive syndrome, or post concussion disorder, is a mild TBI that persists beyond the expected recovery period – more than 3 months. The symptoms are rather nonspecific, subjective symptoms, such as headache, fatigue, dizziness, sleep disturbances, anxiety, irritability and depressed mood [21].

Current research is lacking regarding the management of ice hockey players’ concussions in the long term, and there is limited knowledge on the sustained effects of their injuries. Park *et al.* showed that there is an increasing interest in studying athletes’ career transition out of sport [22]. It was shown that a loss of identity was especially seen when the participants had a strong athletic identity at the end of their sport career. Brewer *et al*. have defined athletic identity as the degree to which an individual identifies with the role of the athlete. According to Ronkainen *et al*., it is a cultural construction produced by dominant discourses [23,24]; in that sense, cultural beliefs and expectations about age, gender, athletic performance and athletic body types all shape understandings of what it means to be an athlete. The aim of this study was to describe what suffering multiple concussions meant for former semi-professional or professional hockey players who were forced to end their career due to concussion.

## Materials & methods

### Participants

The study sample consisted of nine former hockey players – eight men and one woman, all Swedish – who once played on national or professional teams (e.g., in the US National Hockey League) but were forced to quit playing due to suffering multiple concussions. A diagnosis of concussion was given according to the latest diagnostic guidelines on sports-related concussion and players with concussion were managed according to these guidelines [25,26]. The diagnosis of post concussive syndrome was based on DSM-IV criteria [27].

All of the participants had the diagnosed concussions during their hockey career; one of the participants also had another concussion due to a motor vehicle accident. Aged 20–53 years (*Md* = 25), the participants had started playing ice hockey when they were 4–12 years old (*Md* = 6) and quit playing as semi-professionals or professionals when they were 16–37 years old (*Md* = 22). At the time of the study, three participants lived with their parents, three lived alone and three lived with their partners. Two were on sick leave from work, three were studying, two worked as ice hockey trainers, one worked as an ice hockey expert commentator and journalist and one worked as a gardener. At enrollment, the median time since quitting play was 4 years (range, 1–14 years).

### Data collection

Data were collected by the first author during nine qualitative interviews conducted in 2015 [28]. During interviews, participants were asked to narrate their experience of being forced to quit playing hockey due to suffering multiple concussions. Follow-up questions included ‘Please give an example’, ‘How did you feel then?’ and ‘Please describe what you felt’. Each interview lasted 30–80 min and took place over the phone. The interviews were conducted in Swedish and digitally recorded, then transcribed. After this, the interviews were analyzed, and the results were translated into English.

### Ethical considerations

The Regional Ethical Committee approved the study. The Swedish Hockey League was contacted, and letters sent to Swedish ice hockey clubs from which it was known that players had quit due to persistent post concussion symptoms. Ultimately, those players were asked if they were interested in participating our research study. All players provided written informed consent prior to enrollment. Participants were guaranteed anonymity in the presentation of the study findings.

### Phenomenological hermeneutic

Drawing from hermeneutic phenomenology approach, the analysis of the interview transcripts aimed to interpret the transcripts according to a dialectical process that prioritized understanding and explanation [29]. According to Ricoeur [29], the relationship between phenomenology and the hermeneutic philosophy can be accessed to discover the meaning of lived experiences. The interpretation of the transcripts involved three stepwise phases: the formation of a naive understanding, structural analysis and the formation of a discussion and comprehensive understanding [30].

### Losing one’s identity as a hockey player

The primary theme of losing one’s identity as a hockey player was constructed from five sub themes: being limited in everyday life, returning to the hockey stadium as soon as possible, forming a post career identity, finding ways to live a good life and preventing injuries by respecting other players (Table 1).

**Table 1.** Overview of theme (n = 1) and subthemes (n = 5) constructed from the analysis of the interviews.

Theme	Sub theme
Losing one’s identity as a hockey player	Being limited in everyday life Returning to the hockey stadium as soon as possible Forming a post career identity Finding ways to live a good life Preventing injuries by respecting other players

## Results

All participants were successful semi-professional or professional ice hockey players with great ambitions to continue playing at that level, despite having suffered multiple concussions as players. Although they all seemed to understand that the impact of their final concussion was more serious than the effects of previous concussions, the participants nevertheless wanted to return to playing hockey as soon as possible in order to continue being part of their teams and maintain their lives as players. As the leaders of their teams and healthcare professionals struggled to grasp, the gravity of their concussions, participants continued to feel pressured to not quit playing hockey and, as a result, developed more headaches, experienced fatigue and exhibited other cognitive symptoms. In general, the participants experienced being forced to end their careers as ice hockey players as an enormous loss, as playing hockey had been a major part of their lives since childhood.

Informed by the naive understanding, structural analysis resulted in a primary theme with five sub themes, all described and illustrated with quotations from the transcripts in the following subsections.

### Being limited in everyday life

Participants described experiencing headaches every month, if not every day. Living with headaches was unpleasant as well as unpredictable for them, since the origin and intensity of the pain varied. Sometimes, their headaches were extremely debilitating and frightening – as one participant stated, “*like being in hell”*. While recounting experiences with drowsiness and fatigue that persisted even after they had rested, participants also described difficulties with sleeping due to anxiety or pain in the head, jaw or elsewhere in the body. Some also detailed how their personalities had changed and how they had become more impatient and absentminded:

*I have a very short temper. If I feel that I'm not being understood, I lose my patience. I have also heard from my family and friends that my mood is different – I'm more ill-tempered or snappy, more absent, and when the headache increases, I shield myself […] pull away, rest a little bit and try to gather strength.* (participant 3)

Nearly all participants continued to show signs or symptoms of concussions after they had quit playing hockey, except one who had fully recovered from the concussion at the time of the interview. Participants reported that tinnitus, vertigo and increased sensitivity to light, voices and scents made it difficult for them to remain in places with several people at the same time and described avoiding activities and situations that would trigger headaches, as well as using headphones or drinking beer to reduce the effects of babble in crowded places. In those situations, some described almost panicking because they could not determine who said what. In general, the participants expressed frustration with sustaining injuries that influenced their daily lives so considerably, as one memorably described:

*The hardest part is living with a daily impairment. I can't do what I want to do, and it costs so damn much mentally. On the other hand, I feel* *that it's much more difficult to finish things.* (participant 9)

The participants also described feeling a lack of understanding and support from others. They explained how they and others, especially in the beginning of their post injury period, did not view their concussions as serious injuries. Instead, they assumed that, with a couple days of rest and painkillers, the injuries would resolve spontaneously. The invisibility of their injuries prevented others from recognizing their challenges in daily life, especially as professional ice hockey players are generally viewed as healthy and physically fit individuals. Often, participants did not receive effective advice or support from the team leaders, including from coaches, healthcare professionals or social workers:

*I* *never got any advice from physicians or anybody […] The last physician that I had when I played on the team said that he didn't think that [my injury] was a concussion but believed that [the pain] originated in my neck, which made me really pissed […] He couldn't know how or what I felt, but he came out anyway and told the coaches, ‘We have to treat the neck, since the injury is to the neck’, and it immediately became, ‘A-ha, it's just the neck’, and […] ‘He should be back soon’ […] That added some pressure, even though I wasn't feeling well […] and so they checked the calendar and thought, ‘He should back by then [a certain date]’.*” (participant 4)

“*It's been difficult finding the help that I need […] People generally don't really understand; they see [a concussion] as a blow […] but I’ve really felt that it's interfered with my whole personality, and for that, I’ve really wanted help, which I’ve gotten now, at last […] I’ve been a completely different person and had difficulties finding out who I am, what I want and what I can do.* (participant 6)

### Returning to the hockey stadium as soon as possible

After suffering concussions as hockey players, participants recounted how they had missed playing hockey and their teams. They also described their desire to be back on the ice as soon as possible, even though they had nearly fallen asleep or felt dizzy on the bench. The same desire discouraged them from visiting the emergency room directly after experiencing a concussion, for they not only wanted to fulfill their contracts but also feared losing their positions. In participants’ words, ice hockey players who continue to play despite minor injuries and pain are generally appreciated on teams:

*As long as you're not unconscious, you keep playing. It wasn't until last year that the physician said that I’d better take it easy […] I know that he wasn't happy when I played last year. I sometimes couldn't see the puck. During training last year, I would sometimes see three pucks and had a hard time knowing which one to grab. I would go home after each workout and lie down in a dark room, with all of the lights out, but I couldn't manage to hear anything. I couldn't manage to do anything. Doing anything that increased my pulse would immediately give me a splitting headache.*” (participant 5)

“*Hockey began to change a bit after 1989 or 1990. The players started to take better care of their bodies after injuries. Otherwise, there was a bit of a macho culture where you could lie low; you would start playing again as soon as possible. You'd be afraid of losing your position or your job.* (participant 5)

Participants described always feeling indomitable while playing hockey. Although in pain and knowing that playing would make them feel worse, they nevertheless wanted and felt pressured to participate, especially during championships. Even participants who could not train or play while recovering from injuries visited their teams at the ice rink. Some described that their teammates had understood them, since many of them had also suffered concussions and knew how the participants had felt:

*After my third concussion, I played in the U18 World Cup 2 weeks later, and I did it on painkillers because I still had a headache, and I put pressure on myself, but at the same time, I felt pressure from the environment and the team.* (participant 6)

### Forming a post career identity

After suffering their final concussions and discussing their well-being with physicians and their families, participants recognized that they needed to prioritize their health above their careers and decided to quit playing hockey either professionally or on national teams. Invariably, their decisions to stop playing were extremely challenging, if not traumatic, to make; they loved ice hockey, which had been a major part of their lives since childhood and, for most, was also their profession. Although many had not previously considered quitting and were unprepared for such a life-altering transition, they also felt relieved that their struggle to continue playing had ended:

*My decision was more due to [asking myself], ‘Oh, what if I take another blow? What happens after the next injury?’ Making the decision, of course, felt like a very tough call, but there was no doubt. I knew that I wasn't going to play; there was no chance […] Even though I had been completely rehabilitated, I might not recover from the next [concussion]. I have a responsibility to my family. I felt so bad for nearly 2.*5 months*, and I thought, ‘What if I go back and get hit again? Then I might feel this bad again for* 5 months*, or a year, or my whole life.* (participant 4)

Participants described how, after quitting, they had lost their identities as players and did not know who they were, where they should go or what they would do. Some described that ending their lives as semi-professional or professional players had affected their entire families, since living with someone who has been forced to finish playing can be quite demanding. Missing the sport and all that it had given to them, they felt disappointed and often depressed:

*At some point, when I felt that I would never play again, I drank alcohol for 3 weeks straight […] almost immediately after the concussion, because I was really depressed. […] I lost 14 kg in a month, barely ate anything […] I was very sad and disturbed […] mostly because I had dropped out of school during my second year of high school and went to Canada […] It felt like I was empty-handed, without anything, with no card left to play in life*. (participant 6)

After quitting, participants found that meeting former teammates or others, like journalists and fans, interested in their situations was supportive but also stressful. In either case, continuously being approached as an ice hockey player reminded them of what they had lost and made relinquishing their identities as ice hockey players all the more difficult. It was especially disillusioning to realize that, as professional or semi-professional players, they were not indispensable to their teams, especially if their former teammates never contacted them. Participants expressed feeling emptiness every time that a new hockey season started, and that quitting would have been easier on their own terms instead of feeling forced to:

*I didn't decide that I would quit hockey; the physician did. […] I don't think that I’ll ever get over being forced to quit in the middle of the season. […] I’ve had dreams about the last game – the one that I never got to finish […] It's haunted me for years.* (participant 5)

For participants who have had money saved or had adequate health insurance, their post career life has been less challenging. However, this has not been the case for all participants, some of whom became financially dependent on close relatives. Pension funds have supported some participants who became unable to work after their carriers have ended, while others have been left to manage it on their own. As one stated:

*I can't work full-time, and previously I took that for granted. My career path has become very narrow.* (participant 7)

### Finding ways to live a good life

Participants described how support from their families, former teammates and fans had been critical to their transitions out of playing hockey, as well as in various everyday activities, Participants reported that they appreciated their families more than ever before and that social workers and healthcare professionals who had engaged them in supportive dialogue had given them adequate advice and assistance:

*In treatment, I went to a physician and […] I felt like he understood what I meant […] He had a little more commitment […] more advice and tips on things to try, and I also got a daily schedule with some exercises. […] It was also my mom and dad; they were important; […] I got rides to school, and when I was there, they sat waiting in the parking lot […] because [they knew that] I wouldn't be inside very long*. (participant 7)

It was crucial for participants to find meaningful activities immediately after deciding to quit playing hockey, and they described seeking ways to live as normal a life as possible. For many this meant continued involvement in sport and other physical activities. Staying involved with ice hockey in a capacity that did not risk additional injury – for example, attending ice hockey practices and games or working as coaches or equipment managers for other hockey teams – helped them to move forward in their lives. Nevertheless, they struggled to find interests that could replace ice hockey:

*On some days, I feel like I hardly have a future, and on other days, I feel like I want to go and do something. […] It's difficult to say because I feel like […] what will I be able to manage when I’m 30, 40? Will I be better or worse? […] What happens if I take another blow?* (participant 9)

Enjoying the good days and trying to cope with more difficult times, participants described seeking work with flexible hours and workloads as affirming. They wanted to remain optimistic about the future, even if they sometimes experienced anxiety and despair. In general, participants dreamed about becoming better, finding worthwhile activities, becoming able to work full-time and being independent:

*I’ve come to realise that I will live a life with troubles for the rest of my life. It's been* 3 years *now. […] I still oscillate between good and bad days […] and I can recognize the signs that mean, ‘This will probably be a bad day’ […] Bad days eventually become the norm and how my life is. […] I just have to accept it, as I see it. […] Sure, it can be really boring and really hard at times […] but at the same time […] I have the world's absolute best job: a job where I can get some sort of acknowledgement, can do something I’m good at […] and work with the skills that I have.* (participant 4)

### Preventing injuries by respecting other players

To reduce the incidence of injuries among hockey players, participants expressed actively wanting to share their stories and to change aspects of the sport. Reflecting on why the rate of concussions in hockey has risen, despite increased knowledge and better protective equipment, participants explained that everything about the sport – training, games, technology, measurements and pressure – was less intense before. They described that the pace of the game has accelerated, which has reduced the time available to be prepared to protect oneself and avoid being checked on the ice. From their perspective, hockey players today experience greater stress and face greater challenges in being recruited by and staying on professional and semi-professional teams. When every achievement in the game boils down to statistics, when players are constantly compared, when everyone wants to win and when the pressure to perform well is enormous, they explained, players easily overextend themselves on the ice and want to play at the highest level possible as young as they can:

*If you play at a high level and really carry the team in your heart, then you do everything to win the game, and sometimes you do things that aren't so good. You can land wrong while retrieving a puck. There's so much that can happen. You can twist your leg when turning too fast. You're so involved in the sport that you ignore nearly everything that happens to you. And hockey has become much tougher; the bar has been raised a lot over the years.* (participant 2)

As an antidote, participants stressed the importance of recognizing the gravity and prolonged consequences of aggressive concussions and encouraged additional studies on the prevalence of concussions related to ice hockey games. All players, team leaders and coaches need to know the signs of concussive injuries and understand the importance of rest following such injuries. Participants indicated that though such individuals have that knowledge, they do not always apply it but instead believe that it will never happen to them. To raise awareness, they argued, young players in ice hockey schools should be educated early on about proper techniques for checking other players on the ice and protecting themselves:

*Coaches and leaders of younger players […] should teach early on how to both give and receive tackles, because it's a matter of respect* toward *other players. I find it very difficult to believe that a player would hurt someone deliberately. […] So, keeping your elbows down and understanding when and how you can tackle to not hurt anyone or damage anything is important*. (participant 1)

At the same time, players’ parents have a responsibility to ensure that their children receive sufficient rest so that they can truly enjoy playing the game, and it is equally important for young players to be physically fit before participating in higher-level games. Above all, participants emphasized the importance of respecting other players and proposed eliminating every aspect of disrespect and violence in ice hockey. In that sense, they argued that added penalties, new rules or better equipment will not reduce the rate of injuries unless respect for others is prioritized by teams, referees and leaders in the world of hockey:

*I think that maybe there was a little more respect before […] keeping your hands down and not having the elbows up so much. As a coach, you can try to teach ethics – ‘This is how we interpret the rulebook’ – and it's okay to tackle, but we shouldn't do it in a way that isn't fair*. (participant 8)

## Discussion

The loss of personal identity as a hockey player emerged as the primary theme of the interviews with former professional or semi-professional hockey players who sustained multiple concussions during their careers on the ice. By extension, the meanings of athletic identity were rigid and based on physical strength, performance, skills, goal orientation and commitment. Those beliefs and the athletic identity operate as part of the performance narrative that dominates athletic activities and revolves around winning, being superior or achieving other performance-related outcomes [31,32]. Within elite sport organizations and leagues, the performance narrative regularly circulates under the influence of powerful individuals such as coaches, managers and top athletes. Stoltenburg *et al*. suggest that there should be a ‘Plan B’ if athletes suffer a career-ending injury. They also suggest that professionals who work with athletes should prepare all athletes that there will come a day when their sporting career ends [33]. It might be easier to end the career if someone else make the decision.

Participants described experiencing limitations in their everyday lives due to the effects of suffering multiple concussions. Unlike prior to their injuries, they needed to consider not only what they do but also how such activities might affect them – for instance, getting headaches. According to Eriksson, changes in one’s body also changes the person’s access to the world [34]. A comparison of how the body once worked, and now it does not, can thus create a sense of a change in personal identity [35].

However, after sustaining concussions, participants had to somewhat alter their conception of the athletic identity or else lose it entirely. Indeed, as Perrier *et al*. have shown, athletes who acquire physical disability can lose as well as redevelop their athletic identities [36]. Participants expressed missing their pre injury bodies; unable to identify a future for themselves in ice hockey, they began to prioritize the commitment to the sport instead of their level of performance, which helped them to reconstruct their athletic identities post injury with a focus on developing specific skills and simply enjoying the game. Connecting with people who could relate to the experience of suffering from concussions was appreciated.

Given the invisibility of concussion, others often failed to recognize the challenges that participants have experienced in their daily lives. However, developing a coherent understanding of the symptoms and ways to recovery are important following a concussion, this is difficult for sufferers of concussions, regardless of their severity [37]. The invisibility of symptoms endured due to concussions – headaches, for instance – for those without obvious physical impairments undoubtedly contributes to their struggle for validation by others when symptoms persist [38]. Merleau–Ponty points out that the body affords access to the world, meaning that any change in the body changes the experience of the world [39]. Thomas *et al*. have clarified the concept of change in personal identity following TBI by applying concept analysis with loss of self as a label [40]. Among their conclusions, they argue that the more appropriate term following TBI is change in sense of self, defined as “*A change in one’s inner subjective experience as a result of changes in egocentric or socio-centric aspects of self, or in the relationship with one’s identity as shared with others. These changes are of sufficient magnitude that a process of evaluation, acceptance and adaptation is required to regain a unified sense of self”*.

Participants emphasized the importance of having family and friends who understood the prolonged consequences of concussions. Faure and Fitzpatrick examined the lived experiences and attitudes of freestyle BMX and motor-cross athletes after they suffered concussions [41]. Although the athletes in their study accepted the risk of concussion as part of engaging in the sport, they were largely unfamiliar with what concussions are and the possible long-term effects of a history of concussions. Moreover, they were unaware of any protocols in response to concussion sustained in their sport and cited an overall lack of organized medical care accessible to them on an ongoing basis, as is the case with mainstream sports. Nevertheless, they seemed to demonstrate good will toward others by wanting to prevent the effects of concussions that they had experienced, which can be viewed as responding to an ethical demand to care for others’ lives in the best possible way [42]. In light of such results, Batten *et al*. have suggested that attention perhaps should be directed toward preventing athletes from becoming injured in the first place [42].

In the context of the study reported here, to prevent concussions among ice hockey players, the sport should be made structurally safer. To that end, possible means include examining the efficacy of limiting checking exposure during training and games, changing the definition of legal checks, arranging weight- or size-matched groups for children and junior ice hockey leagues and expanding the education of coaches to be more sensitive to concussions, the situations that cause them and ways to respond to them. Such efforts could help to reduce anxiety about concussions, while at the same time encourage the adoption of physically active lifestyles involving athletic activities that are safer for participants [43]. As the pressure to perform well is a part of traditional athletic culture and because high personal value can be gained by players who achieve superior goals, coaches who set those goals and teach ways to accomplish them are important. However, encouraging athletes to develop self-esteem based on self-acceptance, not on achievements or games won, could prove to be a safer strategy for athletes [44]. In relation to this, it is shown that athletes who disrupt their athlete carrier due to retirement, as an expected end, experience less crisis by employing coping strategies for the proactive diminishment of their athletic identities [45].

In professional ice hockey, player-on-player violence and violence remain significant parts of the game [46]. For hockey players, the acceptance of those risks and the willingness to subject the body to injury and pain in order to maintain a personal image of toughness are examples of conventional masculinity. Social norms of the sport are dominated by codes and practices that legitimize such masculinity. Tjønndal writes that the common acceptance, and sometimes encouragement, of player violence and violence against the self in ice hockey has led to many broken bodies, lives and careers among professional athletes [46]. Shedding light on that idea, Ronkainen *et al*. have posited that athletes who cannot meet certain cultural ideals might experience psychological tension and feel less privileged than, as well as socially excluded from, ones who can live up to those ideals [24]. In response, Carless and Douglas have suggested that team managers, coaches, league governing bodies, other athletes and researchers need to support alternative narratives that support athletes’ long-term development and well being, even once their athletic career ends [31,32]. According to Todd *et al.*, the majority of concussions in hockey are caused by body checking [47]. Despite the known negative outcome, there is a resistance to removing body checking from hockey.

That is in line with Cusimano *et al*. who stated that interventions should appeal to young hockey players’ sense of competitiveness and at the same time develop their awareness of injury [48]. This can be done by, for instance, encouraging young players to accept nonviolent role models and to be aware of the serious consequences for aggressive behavior and concussions on the ice. A culture change needs to happen, especially regarding reporting injuries. Cassilo and Sanderson conclude their article by stating: “*understanding that athletes can experience grief-related symptoms as they recover can aid both in supporting athletes as they navigate through this process and in foster cultural change that will encourage more athletes to report concussions, as they will receive empathy and support, rather than pressure and isolation*” [49].

Participants in this study described the support they received from their families, former teammates and fans as critical to their transitioning away from their previous lives as ice hockey players. Stoltenburg *et al*. highlight the importance of social support, especially in the transitioning out of sport [33]. Following the participants’ injuries and departure from the sport as professionals or semi-professionals, they dreamed about becoming better, discovering activities that would give meaning to their lives, becoming able to work full-time and achieving independence. According to Lange *et al.* [50], the presence of depression will significantly increase self-reported post concussions symptoms, in both the absence or presence of a past mild TBI. According to Jones *et al.* [51]. sustaining a head injury does not always lead to deterioration in quality of life, since individuals can be protected from the negative impacts of such injuries by receiving support from social networks and by strengthening their personal identities. For participants, it has been important to stay involved with hockey, even if they could not continue as players.

### Study limitations

The interviews took between 30 and 80 min, which is a rather big difference. However, all participants had experiences they wanted to narrate. Some of them had a lot to tell, while others had less. The interviews were conducted in Swedish and then transcribed and analyzed in Swedish, which might influence some formulations. Nine former hockey players were interviewed in this study, which is a rather small group. The number of interviews required is the number needed to answer the research question [28] and the interviews were rich in content and described similar experiences, which created a pattern that the authors found adequate to serve as a basis for the findings. The small number of participants can therefore be seen as a strength; it gives the opportunity to gain a close and thorough knowledge of those participating in the study.

## Conclusion

Although the former hockey players interviewed seem to have struggled with forming identities and finding sources of meaning for their lives after quitting professional or semi-professional hockey due to suffering multiple concussions, they acknowledged receiving critical support from their families and others in making sense of and managing their daily lives. The players want to share their experiences regarding coping with concussions and extend their knowledge of the injury’s long-term effects in order to prevent other brain injuries among players of hockey, the sport that they continue to love despite the price that they have paid.

## Future perspective

The debate about how to decrease the risks for concussions has to continue. The long-term effect of concussions due to hockey and other sports has to be known by all involved and what can be done for those affected, not only in acute care, but also in everyday life after the hockey career.

**Executive summary**

- The loss of personal identity as a hockey player emerged as the primary theme.
- Given the invisibility of concussion, others often failed to recognize the challenges that participants experienced in their daily lives.
- Participants described experiencing limitations in their everyday lives due to the effects of suffering multiple concussions. They needed to consider not only what they do but also how such activities might affect them.
- Participants emphasized the importance of having family and friends who understood the prolonged consequences of concussions.
- To prevent concussions among ice hockey players, the sport should be made structurally safer and encouraging athletes to develop self-esteem based on self-acceptance could prove to be a safer strategy for athletes.
- For participants, it is important to stay involved with hockey, even if they cannot continue as players.
